# Clinical Profiles, Management, and Outcomes of Complicated Pneumonia in Children: A Retrospective Study from Tertiary Centers in Jordan

**DOI:** 10.3390/diseases13110364

**Published:** 2025-11-08

**Authors:** Lina Alshadfan, Muna Kilani, Saleh Abualhaj, Osama Abu-Salah, Mohammad Ghassab Deameh, Ahmad Nidal Al-Faouri, Mustafa Elayyan, Randa Othman, Reem Abuzraiq

**Affiliations:** 1 Pediatrics and Pediatric Pulmonology, Department of Pediatrics, Faculty of Medicine, Al Balqa Applied University, Al-Salt P.O. Box 19117, Jordan; 2 Pediatrics and Pediatric Pulmonology, Department of Pediatrics, Faculty of Medicine, The Hashemite University, Zarqa P.O. Box 13133, Jordan; mun@hu.edu.jo; 3 General Surgery Department, Faculty of Medicine, Al-Balqa Applied University, Al-Salt P.O. Box 19117, Jordan; 4 Pediatrics, Department of Pediatrics, Faculty of Medicine, Al Balqa Applied University, Al-Salt P.O. Box 19117, Jordan; osamaabusalah@bau.edu.jo (O.A.-S.); randaothman.md@gmail.com (R.O.); reem.abuzraiq@bau.edu.jo (R.A.); 5 Prince Hamzah Hospital, Amman P.O. Box 11947, Jordan; mghdeameh@std.bau.edu.jo (M.G.D.); ahmadalfaouri88@gmail.com (A.N.A.-F.); mustafaelayyan123@gmail.com (M.E.)

**Keywords:** complicated pneumonia, pediatric pneumonia, empyema, necrotizing pneumonia, antibiotic use in children

## Abstract

Background: Complicated pneumonia (CP) in children presents in various forms—including empyema, necrotizing pneumonia (NP), necrotizing pneumonia with pleural effusion (NP + PE), and parapneumonic pleural effusion (PPE)—and is associated with significant morbidity despite advances in antimicrobial therapy. This study aimed to describe and compare the clinical characteristics, laboratory findings, antibiotic use, and outcomes across different CP subtypes in hospitalized children and to assess the impact of prior antibiotic use on presentation and treatment outcomes. Methods: This retrospective observational study included 58 children admitted with CP to tertiary hospitals in Jordan. Patients were categorized into four subtypes: empyema (n = 4), NP (n = 4), NP + PE (n = 17), and PPE (n = 33). Demographic data, clinical features, laboratory results, antibiotic regimens, and clinical outcomes were analyzed. Multivariable regression was used to identify predictors of prior antibiotic use. Results: Fever and cough were the most common symptoms (96.6%). Over 40% of patients had received antibiotics prior to admission. Those pre-treated had significantly longer symptom duration (8.2 vs. 4.5 days, *p* < 0.001), longer hospitalization (18.2 vs. 14.6 days, *p* = 0.023), and more frequent chest tube insertion (66.7% vs. 35.3%, *p* = 0.019). *Streptococcus pneumoniae* was the most common organism isolated in culture-positive cases. Vancomycin-based regimens were the most frequently used treatments. Univariate regression analysis showed that patients with prior antibiotic use had significantly higher odds of longer hospitalization duration (OR = 1.11, *p* = 0.028) and chest tube insertion (OR = 3.67, *p* = 0.021). Conclusions: Complicated pneumonia in children remains a diverse and clinically significant condition. The findings demonstrate that prolonged symptom duration prior to hospitalization and certain clinical interventions were associated with prior antibiotic exposure. These results provide insight into local disease patterns and prescribing behaviors, which may help inform strategies to optimize antimicrobial stewardship and improve care pathways for affected children.

## 1. Introduction

Infectious respiratory diseases (IRDs) remain a major contributor to global morbidity and mortality, particularly among the pediatric population [1], where community-acquired pneumonia (CAP) is a leading cause of hospitalizations and complications worldwide, particularly in low- and middle-income countries [2]. While most cases respond well to appropriate antibiotic therapy, a subset of pediatric patients develop complicated pneumonia (CP)—a severe spectrum of the disease that includes parapneumonic effusion, empyema, and necrotizing pneumonia (NP) [3,4].

While the overall incidence of CAP in children is high, studies report that approximately 3–10% of pediatric pneumonia cases progress to CP [5,6], though rates can vary depending on geographic region, vaccination coverage, and pathogen prevalence [5]. Children at higher risk for developing CP often present with specific characteristics, including younger age (particularly <5 years), delayed presentation to healthcare facilities, incomplete or absent pneumococcal vaccination, underlying chronic illnesses (e.g., congenital heart disease, chronic lung disease, or immunodeficiency), and prior inappropriate or incomplete antibiotic treatment [5,7].

CP represents a complex interplay between pathogen virulence, host immunity, and the adequacy and timing of antibiotic therapy [8]. Empyema and parapneumonic pleural effusions (PPEs) typically result from bacterial invasion of the pleural space following CAP [9], while necrotizing pneumonia involves parenchymal lung destruction, often requiring prolonged therapy or surgical intervention [10]. The coexistence of necrosis and pleural involvement (i.e., NP with effusion) further complicates diagnosis and management [3]. These complications pose significant clinical challenges due to their association with prolonged hospitalizations, increased need for invasive procedures, and intensive antimicrobial therapy, contributing significantly to the healthcare burden [11,12,13].

The incidence and mortality rates of complicated pneumonia (CP) vary according to its subtypes. Parapneumonic effusion (PPE) occurs in about 40% of patients hospitalized with pneumonia, with approximately 5% of hospitalized children developing complicated PPE [6]. Empyema follows with an incidence rate of 2–3% [14], while necrotizing pneumonia (NP) is less common, accounting for 0.8–7% of community-acquired pneumonia (CAP) cases. Despite its lower frequency, NP is often associated with prolonged hospitalization and, in some studies, increased morbidity and mortality [4,15]. Mortality rates also differ among CP subtypes; PPEs requiring pleural drainage carry a mortality rate ranging from 7% to 10%, whereas empyema mortality ranges from 14% to 20% [16].

The most common causative pathogens in complicated pneumonia include *Streptococcus pneumoniae*, *Staphylococcus aureus* (including methicillin-resistant strains), and *Streptococcus pyogenes* [13]. The distribution of these pathogens varies by CP subtype: *S. pneumoniae* is more frequently linked to parapneumonic effusion and empyema, while *S. aureus* and *S. pyogenes* are more often associated with necrotizing pneumonia [17,18,19]. In addition to common bacterial pathogens such as *Streptococcus pneumoniae* and *Staphylococcus aureus*, atypical organisms like *Mycoplasma pneumoniae* and *Mycobacterium tuberculosis* can also cause complicated pneumonia in children [20]. Although these pathogens are less frequently identified in tertiary hospitals in Jordan, they remain clinically important, particularly in cases with prolonged symptoms, poor response to standard antibiotics, or epidemiologic risk factors. Recognizing these causes is essential for timely diagnosis and appropriate management [21,22].

Mortality rates in complicated pneumonia are generally low in high-resource settings but can be affected by the causative pathogen, particularly if antimicrobial resistance is present, as well as by delays or inadequacies in treatment. Resistant pathogens and delayed appropriate therapy are associated with worse outcomes and increased mortality [8,11,23].

Optimal management strategies for CP subtypes remain debated. Controversies exist regarding early chest tube drainage versus conservative management, surgical decortication, or intrapleural fibrinolytics, particularly in resource-limited settings [24,25,26]. Prior antibiotic use before admission may also reduce clinical and microbiological clarity, thereby obscuring pathogen identification and complicating therapeutic decisions [27,28].

The management of complicated pneumonia is further complicated by the rise of antibiotic-resistant pathogens. Inappropriate or empirical overuse of antibiotics in respiratory infections, often in the absence of microbiological confirmation, has fueled the emergence of multidrug-resistant (MDR) organisms [29]. Notably, pathogens such as *Streptococcus pneumoniae*, *Staphylococcus aureus*, and Gram-negative bacilli have demonstrated evolving resistance profiles, including methicillin-resistant *Staphylococcus aureus* (MRSA) and extended-spectrum β-lactamase (ESBL)-producing strains [30]. In Jordan, *S. pneumoniae* and *S. aureus* show distinct epidemiological patterns. *S. pneumoniae* affects both adults and children equally, with isolates primarily from the respiratory tract and blood, and is more prevalent in males. Community-associated MRSA has emerged as the dominant clone in both healthcare and community settings, raising concerns about its growing burden [31]. These trends highlight the importance of targeted infection control and antimicrobial stewardship strategies.

Despite the growing body of literature, data from Middle Eastern and North African (MENA) countries—including Jordan—remain limited, particularly concerning the clinical patterns and antimicrobial management of different subtypes of CP. Understanding these regional differences is essential for developing context-specific clinical guidelines. This retrospective study aims to characterize the clinical presentation and microbiological profiles of children diagnosed with CP, with a particular focus on antibiotic usage patterns and associated outcomes. By identifying current trends in causative organisms and resistance profiles, our findings aim to inform targeted therapy, strengthen infection prevention and control (IPC) practices, and support the broader goal of optimizing antibiotic use in pediatric respiratory infections.

## 2. Materials and Methods

This retrospective observational cohort study was conducted at hospitals affiliated with the Jordanian Ministry of Health (MoH) that have dedicated pediatric departments. The study included all pediatric patients diagnosed and managed for complicated pneumonia between January 2021 and April 2025.

Study Population and Sample

This retrospective study included all pediatric patients (aged 0 to 18 years) diagnosed with complicated community-acquired pneumonia (CP) who were admitted to Ministry of Health (MoH)–related hospitals in Jordan during the study period, using a consensus sampling approach in which all eligible cases were included. Consensus sampling was selected to ensure comprehensive coverage of the study population, avoid sampling bias, and maximize statistical power by utilizing the entire pool of available cases during the defined period.

Patients were classified into four distinct subtypes of CP—empyema, necrotizing pneumonia (NP) without effusion, necrotizing pneumonia with pleural effusion (NP + PE), and parapneumonic pleural effusion (PPE)—based on radiological and clinical findings. Any case that presented with more than one type of CP simultaneously was excluded from the study to ensure accurate classification and analysis. These classifications were confirmed through imaging studies such as chest X-rays, ultrasound, or computed tomography (CT) scans, in conjunction with clinical assessment.

Data Collection

Clinical data were retrieved from hospital electronic medical records using a structured data abstraction form. Collected variables included patient demographics (age, sex), clinical presentation, laboratory parameters (e.g., white blood cell count, CRP), radiological findings, microbiological culture results when available, antibiotic treatment (empiric and definitive), procedural interventions (e.g., thoracentesis, chest tube insertion), length of hospital stay, need for intensive care unit (ICU) admission, and discharge outcomes.

Given the wide variability in weight across pediatric age groups, weight was additionally categorized according to pediatric growth centiles (below the 5th centile or within the normal range) to account for age-related differences and enhance interpretability of weight-related findings.

Laboratory values were collected from the first blood sample obtained at hospital admission. Parameters included white blood cell (WBC) count, platelet count, red blood cell (RBC) count, absolute neutrophil count, lymphocytes, monocytes, eosinophils, basophils, hemoglobin, creatinine, blood urea nitrogen (BUN), and electrolytes (sodium and potassium). Each variable was categorized according to institution-specific pediatric reference ranges. WBC, neutrophils, platelets, and monocytes were classified as high, low, or normal based on age-adjusted limits. Hyponatremia was defined as serum sodium <135 mmol/L, while hypernatremia was >145 mmol/L. Potassium levels were classified as high (>5.0 mmol/L) or normal (3.5–5.0 mmol/L). Similarly, hemoglobin, RBC, and creatinine values were categorized relative to pediatric reference intervals. These standardized thresholds allowed consistent interpretation of laboratory abnormalities across all age groups. As part of the retrospective diagnostic documentation, all patients had been screened for tuberculosis using chest radiography, clinical assessment, and microbiological testing, in accordance with institutional pediatric pneumonia protocols.

Definitions

Complicated pneumonia was defined as pneumonia associated with one or more of the following complications: parapneumonic effusion, empyema, or necrotizing pneumonia. These complications were identified clinically and confirmed by radiological findings.

Empyema: the presence of purulent fluid in the pleural space, confirmed by gross appearance, positive Gram stain, or culture from pleural fluid, in accordance with the British Thoracic Society guidelines.

Parapneumonic pleural effusion (PPE): a pleural effusion associated with pneumonia that is not grossly purulent and does not meet the diagnostic criteria for empyema.

Necrotizing pneumonia (NP): a severe complication of pneumonia characterized by liquefaction and cavitation of lung tissue on imaging, typically confirmed via CT scan, as per pediatric infectious disease consensus criteria.

Ethical Considerations

The study protocol was reviewed and approved by the Institutional Review Board (IRB) of the Al-Balqa Applied University. Ethical approval code: 324/2/3/26. Informed consent was waived due to the retrospective and de-identified nature of the data.

Statistical Analysis

Data were analyzed using R version 4.2.1 (R Foundation for Statistical Computing, Vienna, Austria). Descriptive statistics were used to summarize patient characteristics and outcomes. Continuous variables were expressed as means ± standard deviation (SD) or medians with interquartile range (IQR), depending on data distribution. Categorical variables were summarized as counts and percentages. Comparative analyses were performed using chi-square or Fisher’s exact test for categorical variables and *t*-test or Mann–Whitney U test for continuous variables, as appropriate. Due to the small sample size, no multivariable logistic regression was performed. Instead, associations between prior antibiotic use before admission and selected outcomes (chest tube placement, ICU admission, oxygen therapy, and length of hospitalization) were evaluated using univariate logistic regression models, with results reported as odds ratios (ORs) and 95% confidence intervals (CIs). Covariates were not included in the models due to limited sample size. A *p*-value < 0.05 was considered statistically significant.

## 3. Results

A total of 58 patients met the inclusion criteria. The most common diagnosis was parapneumonic pleural effusion (PPE), accounting for 56.9% (n = 33) of cases, followed by necrotizing pneumonia with pleural effusion (NP + PE) at 29.3% (n = 17). Empyema and necrotizing pneumonia (NP) without pleural effusion were each observed in 6.9% (n = 4) of patients. None of the patients in this cohort had received pneumococcal vaccination, as the pneumococcal conjugate vaccine (PCV) is not part of the national immunization schedule in Jordan.

**Baseline Demographic and Clinical Characteristics by Pneumonia Type**

Empyema (n = 4): Mean age 4.0 years, mean weight 16.8 kg; 50% female. One patient (25%) had chronic conditions/FTT. Symptom duration before admission was longest at 8.2 days. Half received antibiotics prior to admission. Lung involvement was evenly distributed.

Necrotizing Pneumonia (n = 4): Youngest group, mean age 2.5 years, mean weight 11.8 kg; 25% below 5th weight centile. One patient (25%) had chronic conditions; none had prior pneumonia. Mean symptom duration 5.0 days; none received antibiotics before admission. Lung involvement was evenly distributed.

Necrotizing Pneumonia with Pleural Effusion (n = 17): Mean age 3.4 years, mean weight 15.9 kg; 59% male. Prior pneumonia and chronic conditions were present in 35.3% each. Mean symptom duration 6.4 days; 58.8% received antibiotics. Lung involvement was nearly equal.

Parapneumonic Pleural Effusion (n = 33): Mean age 3.3 years, mean weight 15.1 kg; 55% female. Prior pneumonia in 33.3%; chronic conditions in 21.2%. Mean symptom duration 5.7 days; 36.4% received antibiotics. Lung involvement was nearly equal (Table 1).

**Clinical Presentation by CP Type**

Empyema (n = 4): All patients had fever and cough, which was mostly dry. Respiratory compromise was prominent, with 75% showing respiratory distress, hypoxia, and cyanosis, and 100% with tachypnea and retractions. Crackles and reduced breath sounds were universal; dullness to percussion was noted in 25%. Necrotizing Pneumonia (n = 4): Fever was universal, but only half had a cough. Systemic symptoms included decreased oral intake (50%), with no vomiting or hypoactivity. Respiratory severity signs were less frequent; hypoxia and tachypnea were common (75% and 100%). Crackles were present in 75%, and reduced breath sounds in 50%. Necrotizing Pneumonia with Pleural Effusion (n = 17): All had fever and cough (mostly dry, 76.5%), with decreased oral intake (70.6%) and vomiting (23.5%). Respiratory distress and hypoxia were frequent (70.6%), with tachypnea in 88.2%. Crackles (70.6%) and reduced breath sounds (76.5%) were common; dullness to percussion was 11.8%. Parapneumonic Pleural Effusion (n = 33): Fever (93.9%) and cough (100%) were common; decreased oral intake in 60.6%, vomiting in 18.2%, and hypoactivity in 33.3%. Respiratory compromise was less pronounced: respiratory distress 42.4%, hypoxia 66.7%, and cyanosis 39.4%. Crackles in 63.6%; reduced breath sounds in 81.8% (Table 2).

**Labs & Cultures by CP Type**

Empyema (n = 4): All patients had elevated WBC and neutrophils, 75% showed thrombocytosis, and 25% had anemia. Lymphocytes were low in all, and eosinophils were low in 50%. CRP was elevated in all and ESR in 75%. Blood and pleural cultures were negative. Necrotizing Pneumonia (n = 4): Most (75%) had elevated WBC and neutrophils, platelets were normal, and half had positive CRP and ESR. Monocytes, lymphocytes, eosinophils, and RBC counts were generally normal. Cultures were negative. Necrotizing Pneumonia with Pleural Effusion (n = 17): Elevated neutrophils (64.7%) and WBCs (52.9%) were common; 58.8% had thrombocytosis, and about half had anemia (47.1%) and lymphopenia (52.9%). CRP and ESR were positive in 76.5%. Only one patient (5.9%) had positive cultures (*Streptococcus pneumoniae*). Parapneumonic Pleural Effusion (n = 33): High WBC (66.7%), neutrophilia (72.7%), and thrombocytosis (57.6%) were frequent; anemia (60.6%) and lymphopenia (48.5%) were common. CRP was positive in 81.8% and ESR in 60.6%. Blood cultures were positive in 21.2%, mainly *Streptococcus pneumoniae* and *Staphylococcus aureus*. None of the patients had microbiological or clinical evidence of tuberculosis infection (Table 3).

**Antibiotic Use & Treatment Patterns**

Empyema: Half of the patients had prior antibiotics, with a mean duration of 4.2 weeks. In-hospital therapy included vancomycin/meropenem alone (50%) or vancomycin/ceftriaxone followed by piperacillin–tazobactam/amikacin (50%). Post-discharge, 25% received oral clindamycin with amoxicillin–clavulanate, and 75% received amoxicillin–clavulanate alone. NP: None had prior antibiotics; the mean treatment duration was 3.2 weeks. Most received vancomycin/ceftriaxone, then amikacin (50%) or vancomycin/ceftriaxone alone (25%). Post-discharge, half received oral clindamycin plus amoxicillin–clavulanate, and half received amoxicillin–clavulanate alone. NP + PE: Prior antibiotic exposure occurred in 58.8%, with a mean duration of 3.6 weeks. Common regimens were vancomycin/ceftriaxone (29.4%) or sequential vancomycin/ceftriaxone then piperacillin–tazobactam/amikacin (35.3%). About half received oral clindamycin plus amoxicillin–clavulanate at discharge, and 52.9% received amoxicillin–clavulanate alone. PPE: Prior antibiotics were reported in 36.4%, with a mean duration of 3.5 weeks. In-hospital therapy included vancomycin/ceftriaxone (39.4%), vancomycin/meropenem (24.2%), and sequential escalation to piperacillin–tazobactam/amikacin in some cases. Post-discharge, 36.3% received clindamycin plus amoxicillin–clavulanate, 57.6% received amoxicillin–clavulanate alone, and 6.1% received cefixime (Table 4).

Among the total cohort, 24 patients (41.4%) had received antibiotic therapy prior to hospital admission. However, detailed documentation regarding the specific agents used was available for only 22 patients. The most frequently administered pre-hospital antibiotics were amoxicillin/clavulanic acid (n = 8, 13.8%), azithromycin (n = 7, 12.1%), and ceftriaxone (n = 7, 12.1%), while one patient (1.7%) received cefixime (Table 4).

**Clinical Outcomes**

Empyema: Three of four children required ICU admission (mean 5.3 days), all needed oxygen therapy, and half required chest tube insertion (mean 12 days). One patient (25%) underwent VATS. Bronchodilators were used in 50%, and the mean hospital stay was longest at 20.2 days. NP: Most (75%) required ICU care (mean 4.7 days) and oxygen therapy, with no chest tubes or VATS. Bronchodilator use was low (25%), and hospital stay was shortest at 13 days. NP + PE: ICU care was needed in 58.8% (mean 4.5 days), oxygen in 82.4%, and 64.7% required chest tubes (mean 6.2 days). No VATS procedures were performed. Bronchodilator use included salbutamol (41%) and ipratropium (17.6%), with a mean hospital stay of 17.5 days. PPE: ICU admission occurred in 48.5% (mean 5.5 days), oxygen therapy in 57.6%, and chest tube drainage in 45.5% (mean 7.5 days). One patient (3%) underwent VATS. Bronchodilators were used in nearly half (salbutamol 48.5%, ipratropium 24.2%). Mean hospital stay was 15.2 days.

Two patients underwent VATS due to multiloculated empyema and failure of chest tube drainage, despite appropriate medical management, prolonged fever, persistent leukocytosis, and inadequate radiological improvement; both had sterile cultures (Table 5).

None of the patients in this cohort died during hospitalization, and all were discharged in stable clinical condition following completion of treatment.

**Impact of Prior Antibiotic Use on Clinical Presentation and Outcomes**

When comparing children who did and did not receive antibiotics prior to hospital admission, there were no significant differences in age, weight, underlying chronic disease, or previous history of pneumonia. Similarly, radiological patterns (empyema, necrotizing pneumonia, or pleural effusions) did not differ significantly by prior antibiotic use (*p* = 0.14). However, children who had received antibiotics before admission had significantly longer symptom duration prior to hospitalization (mean 8.2 vs. 4.5 days, *p* < 0.001) and longer hospital stays (mean 18.2 vs. 14.6 days, *p* = 0.023). Oxygen therapy was required more frequently in the pre-treated group (83.3% vs. 58.8%, *p* = 0.047). Additionally, chest tube insertion was significantly more common in patients who received antibiotics before admission (66.7% vs. 35.3%, *p* = 0.019). No significant differences were observed in the use of bronchodilators (salbutamol or ipratropium) or surgical intervention (VATS) (Table 6).

**Association Between Prior Antibiotic Use and Clinical Outcomes**

**Figure 1:**
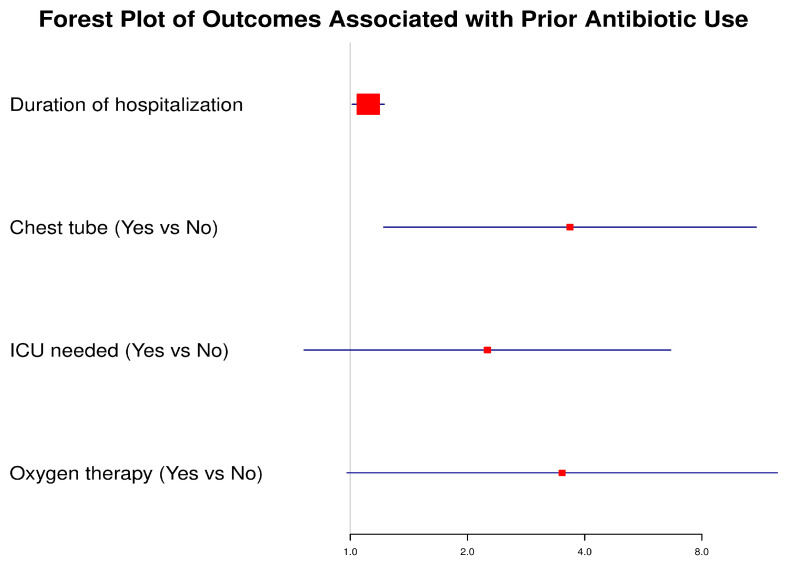
Forest plot of outcomes associated with prior antibiotic use before admission. The plot depicts odds ratios (ORs) and 95% confidence intervals (CIs) for key clinical outcomes. A vertical line at OR = 1 indicates the null effect. Significant associations were observed for chest tube insertion and hospitalization duration, while ICU admission and oxygen therapy showed non-significant trends.

To evaluate the impact of prior antibiotic use before admission on subsequent clinical outcomes, we performed univariate logistic regression analysis. The forest plot (Figure 1) illustrates the odds ratios (ORs) with corresponding 95% confidence intervals (CIs) for selected outcomes. Patients with prior antibiotic use had significantly higher odds of requiring a chest tube (OR: 3.67, 95% CI: 1.22–11.04, *p* = 0.021) and experienced longer hospitalization duration (OR: 1.11, 95% CI: 1.01–1.22, *p* = 0.028). The need for oxygen therapy showed a strong trend toward significance (OR: 3.50, 95% CI: 0.98–12.49, *p* = 0.054). Although patients with prior antibiotic exposure had higher odds of ICU admission (OR: 2.25, 95% CI: 0.76–6.65, *p* = 0.142), this association did not reach statistical significance.

These findings highlight that prior antibiotic exposure may be associated with a more complicated disease course, reflected in higher intervention needs and prolonged hospitalization, though not all associations reached statistical significance.

## 4. Discussion

This retrospective cohort study offers important insights into the clinical presentation, microbiological profiles, management strategies, and outcomes of pediatric patients diagnosed with complicated pneumonia (CP) in Jordan, a lower-middle-income country (LMIC) within the MENA region. Our analysis focused on four clinically significant CP subtypes: empyema, NP, NP with PE, and PPE. Among the 58 children included, PPE was the most prevalent presentation (56.9%), followed by NP with effusion (29.3%), empyema (6.9%), and isolated NP (6.9%). Notably, 41.4% of the patients had received antibiotics prior to hospitalization, which may have contributed to the low microbiological yield (20.3%). *Streptococcus pneumoniae* and *Staphylococcus aureus* were the most frequently identified pathogens. Although most cases were managed medically, 48.3% required surgical intervention either by chest tube or VATS. Empyema was associated with the longest hospital stays and highest complication rates. These findings highlight both the diagnostic and therapeutic complexity of CP and underscore the urgent need for context-specific guidelines and antimicrobial stewardship in pediatric respiratory infections.

Consistent with prior studies, our cohort demonstrated a predominance of pleural space involvement, with PPE being the most frequently observed subtype (56.9%), followed by necrotizing pneumonia with effusion and empyema. This aligns with reports from other LMICs, where delayed presentation, limited access to early imaging, and empirical antibiotic use often lead to progression to complicated pneumonia [32,33].

In our cohort, the mean age of affected children was 3.3 years, with both genders being nearly equally affected by complicated pneumonia. These findings align with global epidemiological trends, which indicate the highest risk among children under five years of age [34,35]. Children in this age group are more susceptible to developing complicated pneumonia due to several factors, including immature immune function, smaller airway diameter, and less efficient mucociliary clearance, all of which predispose them to more severe lower respiratory tract infections [36,37]. Additionally, they are more vulnerable to invasive bacterial pathogens such as *Streptococcus pneumoniae* and *Staphylococcus aureus*, and these infections often progress more rapidly to complications such as parapneumonic effusion or empyema [7].

Systemic features such as fever and decreased oral intake were prevalent across all groups, while respiratory signs—including cough, tachypnea, retractions, hypoxia, and diminished breath sounds—were commonly observed, reflecting extensive pulmonary involvement. These clinical findings are consistent with previously reported patterns in complicated pneumonia, as described by de Benedictis et al. and Chibuk et al., who emphasized the prominence of both systemic and respiratory manifestations in pediatric patients with pleural complications and necrotizing infections [5,38].

Our study represents one of the few from the MENA region to describe the clinical and microbiological characteristics of pediatric complicated pneumonia (CP), and offers a valuable comparison with the recent retrospective cohort by Abdelhady et al. from Egypt, which analyzed 158 pediatric cases of complicated community-acquired pneumonia (cCAP) over five years. While their cohort was larger, our findings share notable similarities. For instance, both studies reported that necrotizing pneumonia (NP) often coexists with pleural complications. In Abdelhady’s study, 64% of NP cases were associated with empyema or pleural effusion, closely mirroring our data in which 17 out of 25 children with NP also had pleural effusion. Additionally, the Egyptian cohort reported low microbiological yield from pleural, sputum, and blood cultures (23%, 18%, and 17%, respectively), which aligns with our study’s similarly limited culture positivity. This underscores the persistent challenge of microbiological diagnosis in CP, often due to prior antibiotic exposure. The positive blood culture rate in this patient population is generally low, even when samples are obtained prior to antibiotic administration. In fact, blood cultures are positive in only a minority of cases (approximately 10–20%), which is aligned with our findings. Nonetheless, it is recommended that blood cultures be collected before initiating antibiotics, as a positive result can help guide targeted antimicrobial therapy [38,39,40].

Furthermore, pleural interventions were common in both studies—87% in Egypt vs. 55.2% in our cohort—though the variation may reflect differences in local protocols or disease severity. Importantly, the Egyptian study highlighted frequent antibiotic modifications (87%), guided largely by clinical response and consultant discretion, a trend also evident in our data. Their use of vancomycin or clindamycin alongside third-generation cephalosporins as first-line empiric therapy was similar to our center’s approach, though broader-spectrum agents like meropenem and linezolid were also employed later, raising stewardship concerns. In our study, these regimens were selected to provide broad-spectrum coverage for the most common causative pathogens associated with complicated pneumonia in children. Vancomycin was included to target methicillin-resistant *Staphylococcus aureus* (MRSA), while ceftriaxone offered coverage for *Streptococcus pneumoniae*—including many penicillin-resistant strains—and common Gram-negative organisms. Sequential combinations such as piperacillin–tazobactam with amikacin were employed to extend coverage to Gram-negative bacilli, including Pseudomonas aeruginosa, and to address potential polymicrobial infections in severe or hospital-acquired cases. Finally, while Abdelhady et al. reported significant morbidity—including PICU admissions (29%) and thoracostomy tube insertion (41%)—our cohort demonstrated even higher rates, with 55.2% requiring ICU care and 48.3% undergoing chest tube placement. These differences may reflect variations in disease severity, case definitions, referral patterns, or the timing of diagnosis and intervention. Collectively, this comparison emphasizes the shared regional burden of complicated pneumonia in children and underscores the urgent need for updated, regionally adapted management guidelines and improved microbiological diagnostic capacity [3].

Our findings also suggest that certain clinical parameters may correlate with disease severity. Patients with necrotizing pneumonia and empyema were more likely to present with severe leukocytosis, anemia, and prolonged hospitalization, reinforcing prior evidence that necrosis and purulent pleural collections signify advanced disease stages [15,41]. However, due to the small sample size, we were unable to perform robust multivariable analysis to define predictors of outcomes.

One of the most novel and clinically significant findings of our study was the investigation of pre-admission antibiotic use, which was reported in nearly 41% of cases. This factor emerged as a potential contributor to disease complexity and poorer clinical trajectories. Patients who had received antibiotics prior to hospitalization exhibited more severe clinical courses: they presented with a longer duration of symptoms before admission, prolonged hospital stays, and a higher likelihood of requiring oxygen therapy, chest tube insertion, and surgical intervention. Notably, these patients also had a greater incidence of necrotizing pneumonia with pleural effusion, suggesting that early partial treatment may have masked the typical clinical presentation, delayed diagnosis, or contributed to disease progression [42,43]. These findings emphasize the critical implications of early outpatient management and highlight the potential harm of incomplete or inappropriate antibiotic regimens, particularly in resource-limited settings where close follow-up and early imaging are not always feasible [44,45]. Several factors may contribute to inappropriate antibiotic use in the pre-hospital setting within our healthcare system. These include the absence or limited dissemination of national guidelines for pediatric respiratory infections, incomplete knowledge of the local prevalence of causative pulmonary pathogens in community-acquired infections, and gaps in antimicrobial stewardship (ABS) skills among primary care pediatricians [46,47,48].

Moreover, the limited response to pre-admission antibiotic therapy in our cohort may also reflect the growing burden of antimicrobial resistance (AMR) in Jordan. Recent evaluations of the National Action Plan on Antimicrobial Resistance (2018–2022) revealed persistent challenges in stewardship implementation, surveillance coverage, and diagnostic infrastructure [49]. These systemic barriers, combined with empiric antibiotic use in outpatient settings without microbiological confirmation, likely contribute to the reduced effectiveness of empirical regimens and the progression of community-acquired infections.

In resource-limited settings, additional challenges such as limited access to diagnostic imaging, lack of timely follow-up, and pressure from caregivers to prescribe antibiotics can further exacerbate inappropriate use [50]. Moreover, the effect of pre-admission antibiotics may help explain the high rate of culture negativity observed in our study. Sputum/pleural fluid cultures were negative in up to 93% of cases, and blood cultures were negative in 86.2%, a pattern that significantly limits the identification of causative pathogens and impairs targeted antibiotic therapy. Early empirical antibiotic use likely suppressed bacterial growth, rendering cultures less sensitive and complicating both diagnosis and treatment [51,52]. This reinforces the importance of capturing a detailed antibiotic history on presentation and highlights the need for alternative diagnostic approaches to improve etiologic detection in antibiotic-pretreated children.

From a public health standpoint, our study highlights the importance of early detection, appropriate antibiotic initiation, and access to radiological imaging and surgical services in mitigating pneumonia complications. These challenges remain particularly relevant in LMIC settings, where resource constraints may delay optimal care. Avoiding unnecessary empiric antibiotic use before adequate clinical and radiological evaluation—particularly in primary care and community settings—may help prevent masking of typical clinical features, reduce diagnostic delays, and limit progression to complicated pneumonia [53,54]. Strengthening adherence to established pediatric pneumonia guidelines that emphasize appropriate diagnostic assessment prior to initiating antibiotics, ensuring timely referral of children with persistent or severe respiratory symptoms to hospital care, and promoting clinician education on early warning signs of complicated pneumonia can collectively reduce premature or inappropriate antibiotic use and facilitate earlier, targeted interventions [55].

It is noteworthy that none of the included patients had received pneumococcal vaccination, as PCV is not part of the national immunization program in Jordan. This may partially explain the continued burden of complicated bacterial pneumonia among children. Strengthening vaccination coverage, especially pneumococcal conjugate vaccines (PCV), and reinforcing antimicrobial stewardship programs could significantly reduce the incidence of severe pneumonia and its complications [56,57,58].

Our study has several notable strengths. The use of detailed and systematically collected data allowed for comprehensive analysis of clinical, radiological, and management-related variables. The inclusion of a well-defined pediatric cohort with radiologically confirmed pneumonia subtypes added clarity to our classification and strengthened the validity of subgroup comparisons.

However, some limitations must be acknowledged. The study’s retrospective design and relatively small sample size may limit the statistical power and generalizability of the findings, particularly when extrapolating to other populations with different healthcare systems or epidemiological profiles. The microbiological diagnostic yield was low, likely influenced by prior antibiotic use, and we were unable to evaluate antimicrobial resistance patterns due to the lack of susceptibility data. Additionally, long-term outcomes such as pulmonary function, recurrence, or quality of life post-discharge were not assessed. The absence of standardized radiological review protocols may have also introduced classification bias across pneumonia subtypes.

Looking ahead, future research in larger, multicenter, and prospective cohorts is needed to confirm the reliability of these findings in diverse settings, with a particular focus on investigating whether the use of antibiotics prior to admission is a significant predictor of worse outcomes. Incorporating molecular or rapid diagnostic technologies could enhance pathogen detection, particularly in pretreated cases. Further studies should also explore interventions aimed at improving outpatient antibiotic stewardship and evaluate the impact of adjunctive therapies on reducing hospital stay, complications, and long-term outcomes.

## 5. Conclusions

In conclusion, complicated pneumonia remains a significant pediatric health concern, particularly in resource-limited settings. Our data underscore the clinical diversity of these cases, the changing microbiological spectrum, and the importance of early multidisciplinary management. Our findings also highlight the importance of considering pre-admission antibiotic exposure, which may mask typical clinical features and contribute to disease progression.

While these findings provide valuable insights into real-world management and antibiotic use in the absence of standardized stewardship programs, they should be interpreted cautiously given the relatively small sample size and retrospective design. Nonetheless, they highlight the need to strengthen adherence to pediatric pneumonia guidelines, ensure timely hospital referral for severe cases, and reinforce infection prevention and control (IPC) measures to reduce complications.

Future multicenter studies incorporating prospective designs, microbial resistance profiling, and long-term outcomes are needed to guide tailored interventions and reduce the burden of pediatric respiratory infections.

## Figures and Tables

**Table 1:** Baseline Demographic and Clinical Characteristics of Children with Complicated Pneumonia, by Pneumonia Type.

	Empyema (N = 4)	NP (N = 4)	NP + PE (N = 17)	PPE (N = 33)	Total (N = 58)
Age (years)					
Mean (SD)	4.0 (2.3)	2.5 (2.6)	3.4 (1.4)	3.3 (1.7)	3.3 (1.7)
Range	1.9–6.0	0.2–5.0	2.0–6.0	1.0–8.0	0.2–8.0
Weight (kg)					
Mean (SD)	16.8 (6.9)	11.8 (7.8)	15.9 (3.2)	15.1 (3.2)	15.2 (3.9)
Range	11.0–26.0	4.5–19.0	12.0–24.0	10.0–20.0	4.5–26.0
Weight Centile					
Below 5th	0.0 (0.0%)	1.0 (25.0%)	0.0 (0.0%)	3.0 (9.1%)	4.0 (6.9%)
Normal	4.0 (100.0%)	3.0 (75.0%)	17.0 (100.0%)	30.0 (90.9%)	54.0 (93.1%)
Gender					
Female	2.0 (50.0%)	2.0 (50.0%)	7.0 (41.2%)	18.0 (54.5%)	29.0 (50.0%)
Male	2.0 (50.0%)	2.0 (50.0%)	10.0 (58.8%)	15.0 (45.5%)	29.0 (50.0%)
Previous History of Pneumonia					
No	3.0 (75.0%)	4.0 (100.0%)	11.0 (64.7%)	22.0 (66.7%)	40.0 (69.0%)
Yes	1.0 (25.0%)	0.0 (0.0%)	6.0 (35.3%)	11.0 (33.3%)	18.0 (31.0%)
Chronic Diseases (or Failure to Thrive)					
No	3.0 (75.0%)	3.0 (75.0%)	11.0 (64.7%)	26.0 (78.8%)	43.0 (74.1%)
Yes	1.0 (25.0%)	1.0 (25.0%)	6.0 (35.3%)	7.0 (21.2%)	15.0 (25.9%)
Duration of Symptoms Before Admission (days)					
Mean (SD)	8.2 (5.4)	5.0 (2.9)	6.4 (3.5)	5.7 (3.2)	6.0 (3.4)
Range	3.0–15.0	2.0–8.0	2.0–14.0	2.0–14.0	2.0–15.0
Antibiotic Use before Admission					
No	2.0 (50.0%)	4.0 (100.0%)	7.0 (41.2%)	21.0 (63.6%)	34.0 (58.6%)
Yes	2.0 (50.0%)	0.0 (0.0%)	10.0 (58.8%)	12.0 (36.4%)	24.0 (41.4%)
Affected Lung Side					
Left	2.0 (50.0%)	2.0 (50.0%)	9.0 (52.9%)	16.0 (48.5%)	29.0 (50.0%)
Right	2.0 (50.0%)	2.0 (50.0%)	8.0 (47.1%)	17.0 (51.5%)	29.0 (50.0%)

**Table 2:** Clinical Signs and Symptoms of Children with Complicated Pneumonia.

	Signs and Symptoms	Empyema (N = 4)	NP (N = 4)	NP + PE (N = 17)	PPE (N = 33)	Total (N = 58)
Systemic manifestations	Fever	4.0 (100.0%)	4.0 (100.0%)	17.0 (100.0%)	31.0 (93.9%)	56.0 (96.6%)
	Vomiting	0.0 (0.0%)	0.0 (0.0%)	4.0 (23.5%)	6.0 (18.2%)	10.0 (17.2%)
	Decrease oral intake	2.0 (50.0%)	2.0 (50.0%)	12.0 (70.6%)	20.0 (60.6%)	36.0 (62.1%)
	Hypoactivity	1.0 (25.0%)	0.0 (0.0%)	6.0 (35.3%)	11.0 (33.3%)	18.0 (31.0%)
Respiratory complaints	Cough	4.0 (100.0%)	2.0 (50.0%)	17.0 (100.0%)	33.0 (100.0%)	56.0 (96.6%)
	Dry cough	3.0 (75.0%)	1.0 (25.0%)	13.0 (76.5%)	23.0 (69.7%)	40.0 (69.0%)
	Wet cough	2.0 (50.0%)	1.0 (25.0%)	8.0 (47.1%)	13.0 (39.4%)	24.0 (41.4%)
	Hemoptysis	0.0 (0.0%)	0.0 (0.0%)	1.0 (5.9%)	1.0 (3.0%)	2.0 (3.4%)
	SOB	3.0 (75.0%)	1.0 (25.0%)	8.0 (47.1%)	10.0 (30.3%)	22.0 (37.9%)
Severity signs	respiratory distress	3.0 (75.0%)	1.0 (25.0%)	12.0 (70.6%)	14.0 (42.4%)	30.0 (51.7%)
	Hypoxia	3.0 (75.0%)	3.0 (75.0%)	12.0 (70.6%)	22.0 (66.7%)	40.0 (69.0%)
	Cyanosis	3.0 (75.0%)	1.0 (25.0%)	5.0 (29.4%)	13.0 (39.4%)	22.0 (37.9%)
	Tachypnea	4.0 (100.0%)	4.0 (100.0%)	15.0 (88.2%)	27.0 (81.8%)	50.0 (86.2%)
	Retractions	4.0 (100.0%)	3.0 (75.0%)	14.0 (82.4%)	25.0 (75.8%)	46.0 (79.3%)
Physical exam signs	crackles	4.0 (100.0%)	3.0 (75.0%)	12.0 (70.6%)	21.0 (63.6%)	40.0 (69.0%)
	Dullness to percussion	1.0 (25.0%)	0.0 (0.0%)	2.0 (11.8%)	3.0 (9.1%)	6.0 (10.3%)
	Reduced breath sounds	4.0 (100.0%)	2.0 (50.0%)	13.0 (76.5%)	27.0 (81.8%)	46.0 (79.3%)
	Tachycardia	0.0 (0.0%)	1.0 (25.0%)	3.0 (17.6%)	4.0 (12.1%)	8.0 (13.8%)

**Table 3:** Laboratory and Microbiological Findings in Children with Complicated Pneumonia.

	Empyema (N = 4)	NP (N = 4)	NP + PE (N = 17)	PPE (N = 33)	Total (N = 58)
WBC					
High	4.0 (100.0%)	3.0 (75.0%)	9.0 (52.9%)	22.0 (66.7%)	38.0 (65.5%)
Low	0.0 (0.0%)	0.0 (0.0%)	0.0 (0.0%)	2.0 (6.1%)	2.0 (3.4%)
Normal	0.0 (0.0%)	1.0 (25.0%)	8.0 (47.1%)	9.0 (27.3%)	18.0 (31.0%)
Platelet Count					
High	3.0 (75.0%)	0.0 (0.0%)	10.0 (58.8%)	19.0 (57.6%)	32.0 (55.2%)
Normal	1.0 (25.0%)	4.0 (100.0%)	7.0 (41.2%)	14.0 (42.4%)	26.0 (44.8%)
RBC					
Low	1.0 (25.0%)	0.0 (0.0%)	4.0 (23.5%)	7.0 (21.2%)	12.0 (20.7%)
Normal	3.0 (75.0%)	4.0 (100.0%)	13.0 (76.5%)	26.0 (78.8%)	46.0 (79.3%)
Neutrophils					
High	4.0 (100.0%)	3.0 (75.0%)	11.0 (64.7%)	24.0 (72.7%)	42.0 (72.4%)
Normal	0.0 (0.0%)	1.0 (25.0%)	6.0 (35.3%)	9.0 (27.3%)	16.0 (27.6%)
Monocytes					
High	1.0 (25.0%)	1.0 (25.0%)	3.0 (17.6%)	11.0 (33.3%)	16.0 (27.6%)
Low	1.0 (25.0%)	0.0 (0.0%)	2.0 (11.8%)	5.0 (15.2%)	8.0 (13.8%)
Normal	2.0 (50.0%)	3.0 (75.0%)	12.0 (70.6%)	17.0 (51.5%)	34.0 (58.6%)
Lymphocytes					
Low	4.0 (100.0%)	1.0 (25.0%)	9.0 (52.9%)	16.0 (48.5%)	30.0 (51.7%)
Normal	0.0 (0.0%)	3.0 (75.0%)	8.0 (47.1%)	17.0 (51.5%)	28.0 (48.3%)
Eosinophils					
Low	2.0 (50.0%)	0.0 (0.0%)	0.0 (0.0%)	6.0 (18.2%)	8.0 (13.8%)
Normal	2.0 (50.0%)	4.0 (100.0%)	17.0 (100.0%)	27.0 (81.8%)	50.0 (86.2%)
Hemoglobin					
Low	1.0 (25.0%)	1.0 (25.0%)	8.0 (47.1%)	20.0 (60.6%)	30.0 (51.7%)
Normal	3.0 (75.0%)	3.0 (75.0%)	9.0 (52.9%)	13.0 (39.4%)	28.0 (48.3%)
Basophils					
High	1.0 (25.0%)	0.0 (0.0%)	0.0 (0.0%)	3.0 (9.1%)	4.0 (6.9%)
Low	2.0 (50.0%)	1.0 (25.0%)	3.0 (17.6%)	4.0 (12.1%)	10.0 (17.2%)
Normal	1.0 (25.0%)	3.0 (75.0%)	14.0 (82.4%)	26.0 (78.8%)	44.0 (75.9%)
Creatinine					
High	0.0 (0.0%)	0.0 (0.0%)	1.0 (5.9%)	1.0 (3.0%)	2.0 (3.4%)
Low	1.0 (25.0%)	1.0 (25.0%)	3.0 (17.6%)	7.0 (21.2%)	12.0 (20.7%)
Normal	3.0 (75.0%)	3.0 (75.0%)	13.0 (76.5%)	25.0 (75.8%)	44.0 (75.9%)
Blood Urea Nitrogen (BUN)					
Low	1.0 (25.0%)	0.0 (0.0%)	3.0 (17.6%)	4.0 (12.1%)	8.0 (13.8%)
Normal	3.0 (75.0%)	4.0 (100.0%)	14.0 (82.4%)	29.0 (87.9%)	50.0 (86.2%)
Na Level					
High	0.0 (0.0%)	0.0 (0.0%)	1.0 (5.9%)	1.0 (3.0%)	2.0 (3.4%)
Low	1.0 (25.0%)	1.0 (25.0%)	5.0 (29.4%)	7.0 (21.2%)	14.0 (24.1%)
Normal	3.0 (75.0%)	3.0 (75.0%)	11.0 (64.7%)	25.0 (75.8%)	42.0 (72.4%)
K Level					
High	0.0 (0.0%)	0.0 (0.0%)	0.0 (0.0%)	2.0 (6.1%)	2.0 (3.4%)
Normal	4.0 (100.0%)	4.0 (100.0%)	17.0 (100.0%)	31.0 (93.9%)	56.0 (96.6%)
C-Reactive Protein (CRP)					
Negative	0.0 (0.0%)	2.0 (50.0%)	4.0 (23.5%)	6.0 (18.2%)	12.0 (20.7%)
Positive	4.0 (100.0%)	2.0 (50.0%)	13.0 (76.5%)	27.0 (81.8%)	46.0 (79.3%)
Erythrocyte Sedimentation Rate (ESR)					
Negative	1.0 (25.0%)	2.0 (50.0%)	4.0 (23.5%)	13.0 (39.4%)	20.0 (34.5%)
Positive	3.0 (75.0%)	2.0 (50.0%)	13.0 (76.5%)	20.0 (60.6%)	38.0 (65.5%)
Pleural Fluid/Sputum Culture					
Negative	4.0 (100.0%)	4.0 (100.0%)	16.0 (94.1%)	30.0 (90.9%)	54.0 (93.1%)
Positive	0.0 (0.0%)	0.0 (0.0%)	1.0 (5.9%)	3.0 (9.1%)	4.0 (6.9%)
Culture-Blood					
Negative	4.0 (100.0%)	4.0 (100.0%)	16.0 (94.1%)	26.0 (78.8%)	50.0 (86.2%)
Positive	0.0 (0.0%)	0.0 (0.0%)	1.0 (5.9%)	7.0 (21.2%)	8.0 (13.8%)
If Positive, Name of The Organism (Specify)					
*S. aureus*	0	0	0.0 (0.0%)	3.0 (37.5%)	3.0 (30.0%)
Skin Contamination	0	0	0.0 (0.0%)	1.0 (12.5%)	1.0 (10.0%)
*S. pneumoniae*	0	0	2.0 (100.0%)	4.0 (50.0%)	6.0 (60.0%)

**Table 4:** Antibiotic Use Before Admission, In-Hospital Regimens, and Post-Discharge Oral Therapy Among Children with Complicated Pneumonia (N = 58).

	Empyema (N = 4)	NP (N = 4)	NP + PE (N = 17)	PPE (N = 33)	Total (N = 58)
Antibiotic used before admission					
No	2.0 (50.0%)	4.0 (100.0%)	7.0 (41.2%)	21.0 (63.6%)	34.0 (58.6%)
Yes	2.0 (50.0%)	0.0 (0.0%)	10.0 (58.8%)	12.0 (36.4%)	24.0 (41.4%)
Antibiotic used before admission—Type					
Amoxicillin/clavulanic acid	1.0 (25.0%)	0.0 (0.0%)	3.0 (17.6%)	4.0 (12.1%)	8.0 (13.8%)
Azithromycin	1.0 (25.0%)	0.0 (0.0%)	1.0 (5.9%)	5.0 (15.2%)	7.0 (12.1%)
Ceftriaxone	0.0 (0.0%)	0.0 (0.0%)	5.0 (29.4%)	2.0 (6.1%)	7.0 (12.1%)
Cefixime	0.0 (0.0%)	0.0 (0.0%)	0.0 (0.0%)	1.0 (3.0%)	1.0 (1.7%)
Overall antibiotics duration weeks					
Mean (SD)	4.2 (1.7)	3.2 (0.5)	3.6 (1.1)	3.5 (0.9)	3.5 (1.0)
Range	2.0–6.0	3.0–4.0	2.0–6.0	2.0–6.0	2.0–6.0
Antibiotics administered during hospitalization					
Vancomycin and Meropenem only	2.0 (50.0%)	0.0 (0.0%)	2.0 (11.8%)	8.0 (24.2%)	12.0 (20.7%)
Vancomycin and Ceftriaxone only	0.0 (0.0%)	1.0 (25.0%)	5.0 (29.4%)	13.0 (39.4%)	19.0 (32.8%)
Piperacillin/Tazobactam and Amikacin only	0.0 (0.0%)	0.0 (0.0%)	0.0 (0.0%)	4.0 (12.1%)	4.0 (6.9%)
Vancomycin and Meropenem then Piperacillin/Tazobactam and Amikacin	0.0 (0.0%)	0.0 (0.0%)	4.0 (23.5%)	1.0 (3.0%)	5.0 (8.6%)
Vancomycin and Ceftriaxone then Amikacin	0.0 (0.0%)	2.0 (50.0%)	0.0 (0.0%)	0.0 (0.0%)	2.0 (3.4%)
Vancomycin and Ceftriaxone then Piperacillin/Tazobactam and Amikacin	2.0 (50.0%)	1.0 (25.0%)	6.0 (35.3%)	7.0 (21.2%)	16.0 (27.6%)
Oral antibiotics after discharge					
Clindamycin + Amoxicillin–clavulanate	1.0 (25.0%)	2.0 (50.0%)	8.0 (47.1%)	12.0 (36.3%)	23.0 (39.7%)
Amoxicillin–clavulanate alone	3.0 (75.0%)	2.0 (50.0%)	9.0 (52.9%)	19.0 (57.6%)	33.0 (56.9%)
Cefixime	0	0	0	2.0 (6.1%)	2 (3.4%)

**Table 5:** Clinical Outcomes and Interventions for Children with Complicated Pneumonia (N = 58).

	Empyema (N = 4)	NP (N = 4)	NP + PE (N = 17)	PPE (N = 33)	Total (N = 58)
ICU admission needed					
No	1.0 (25.0%)	1.0 (25.0%)	7.0 (41.2%)	17.0 (51.5%)	26.0 (44.8%)
Yes	3.0 (75.0%)	3.0 (75.0%)	10.0 (58.8%)	16.0 (48.5%)	32.0 (55.2%)
If admitted to ICU, duration in ICU (days)					
Mean (SD)	5.3 (0.6)	4.7 (1.5)	4.5 (1.5)	5.5 (2.1)	5.1 (1.8)
Range	5.0–6.0	3.0–6.0	3.0–7.0	3.0–10.0	3.0–10.0
Oxygen therapy					
No	0.0 (0.0%)	1.0 (25.0%)	3.0 (17.6%)	14.0 (42.4%)	18.0 (31.0%)
Yes	4.0 (100.0%)	3.0 (75.0%)	14.0 (82.4%)	19.0 (57.6%)	40.0 (69.0%)
Bronchodilators [Salbutamol]					
No	2.0 (50.0%)	3.0 (75.0%)	10.0 (58.8%)	17.0 (51.5%)	32.0 (55.2%)
Yes	2.0 (50.0%)	1.0 (25.0%)	7.0 (41.2%)	16.0 (48.5%)	26.0 (44.8%)
Bronchodilators [Ipratropium]					
No	4.0 (100.0%)	3.0 (75.0%)	14.0 (82.4%)	25.0 (75.8%)	46.0 (79.3%)
Yes	0.0 (0.0%)	1.0 (25.0%)	3.0 (17.6%)	8.0 (24.2%)	12.0 (20.7%)
Surgical intervention [VATS]					
No	3.0 (75.0%)	4.0 (100.0%)	17.0 (100.0%)	32.0 (97.0%)	56.0 (96.6%)
Yes	1.0 (25.0%)	0.0 (0.0%)	0.0 (0.0%)	1.0 (3.0%)	2.0 (3.4%)
Surgical intervention [Chest tube]					
No	2.0 (50.0%)	4.0 (100.0%)	6.0 (35.3%)	18.0 (54.5%)	30.0 (51.7%)
Yes	2.0 (50.0%)	0.0 (0.0%)	11.0 (64.7%)	15.0 (45.5%)	28.0 (48.3%)
If chest tube inserted, duration of chest tube days					
Mean (SD)	12.0 (0.0)	NA	6.2 (4.2)	7.5 (3.4)	7.3 (3.8)
Range	12.0–12.0	NA	3.0–17.0	4.0–16.0	3.0–17.0
Duration of hospitalization (day)					
Mean (SD)	20.2 (5.9)	13.0 (1.8)	17.5 (5.9)	15.2 (6.2)	16.1 (6.0)
Range	14.0–28.0	11.0–15.0	7.0–27.0	6.0–28.0	6.0–28.0

**Table 6:** Bivariate Comparison of Children With and Without Prior Antibiotic Use Before Admission (N = 58).

	Did Not Use Antibiotic Prior Admission (N = 34, 58.6%)	Used (N = 24, 41.4%)	*p* Value
Age (year)			0.700 ^1^
Mean (SD)	3.4 (1.9)	3.2 (1.4)	
Range	0.2–8.0	1.8–6.0	
Weight kg			0.883 ^1^
Mean (SD)	15.2 (3.8)	15.3 (4.2)	
Range	4.5–20.0	10.0–26.0	
Type of Complicated Pneumonia			0.143 ^2^
Empyema	2.0 (5.9%)	2.0 (8.3%)	
NP	4.0 (11.8%)	0.0 (0.0%)	
NP + PE	7.0 (20.6%)	10.0 (41.7%)	
PPE	21.0 (61.8%)	12.0 (50.0%)	
Previous Hx of Pneumonia			0.404 ^2^
No	22.0 (64.7%)	18.0 (75.0%)	
Yes	12.0 (35.3%)	6.0 (25.0%)	
Chronic Diseases, FTT			0.900 ^2^
No	25.0 (73.5%)	18.0 (75.0%)	
Yes	9.0 (26.5%)	6.0 (25.0%)	
Duration of symptom before admission(days)			<0.001 ^1^
Mean (SD)	4.5 (2.3)	8.2 (3.6)	
Range	2.0–11.0	3.0–15.0	
Duration of hospitalization (days)			0.023 ^1^
Mean (SD)	14.6 (4.9)	18.2 (6.8)	
Range	7.0–27.0	6.0–28.0	
Oxygen therapy			0.047 ^2^
No	14.0 (41.2%)	4.0 (16.7%)	
Yes	20.0 (58.8%)	20.0 (83.3%)	
Intervention [VATS]			0.227 ^2^
No	32.0 (94.1%)	24.0 (100.0%)	
Yes	2.0 (5.9%)	0.0 (0.0%)	
Bronchodilators [Salbutamol]			0.684 ^2^
No	18.0 (52.9%)	14.0 (58.3%)	
Yes	16.0 (47.1%)	10.0 (41.7%)	
Bronchodilators [Ipratropium]			0.525 ^2^
No	26.0 (76.5%)	20.0 (83.3%)	
Yes	8.0 (23.5%)	4.0 (16.7%)	
Intervention [Chest tube]			0.019 ^2^
No	22.0 (64.7%)	8.0 (33.3%)	
Yes	12.0 (35.3%)	16.0 (66.7%)	

*p*-values are from ^1^ *t*-tests for continuous variables and ^2^ chi-square tests for categorical variables.

## Data Availability

The data underlying this article are available in the article and upon request from the corresponding author: the data are not publicly available due to privacy or institutional regulations.

## Abbreviations

The following abbreviations are used in this manuscript:

AMR	Antimicrobial Resistance
AST	Antimicrobial Susceptibility Testing
BUN	Blood Urea Nitrogen
CAP	Community-Acquired Pneumonia
CP	Complicated Pneumonia
CRP	C-Reactive Protein
CT	Computed Tomography
CXR	Chest X-ray
ESBL	Extended-Spectrum Beta-Lactamase
ESR	Erythrocyte Sedimentation Rate
FTT	Failure To Thrive
ICU	Intensive Care Unit
IPC	Infection Prevention and Control
IRDs	Infectious Respiratory Diseases
K	Potassium level
LMIC	Lower-Middle-Income Country
LOS	Length of Stay
MDR	Multidrug-Resistant
MENA	Middle East and North Africa
MoH	Ministry of Health
MRSA	*Methicillin-Resistant Staphylococcus aureus*
Na	Sodium level
NP	Necrotizing Pneumonia
PE	Pleural Effusion
PPE	Parapneumonic Pleural Effusion
*S. aureus*	*Staphylococcus aureus*
*S. pneumoniae*	*Streptococcus pneumoniae*
SOB	Shortness Of Breath
VATS	Video-Assisted Thoracoscopic Surgery
WBC	White Blood Cell count
